# Single-cell RNA sequencing reveals a fibroblast gene signature that promotes T-cell infiltration in muscle-invasive bladder cancer

**DOI:** 10.1038/s42003-025-08094-9

**Published:** 2025-05-03

**Authors:** Zige Liu, Xingning Mao, Yuli Xie, Yunkun Yan, Xiang Wang, Junhao Mi, Hao Yuan, Jiange Zhang, Caisheng Huang, Jianxin Chen, Mujia Jili, Shengzhu Huang, Qingyun Zhang, Fubo Wang, Zengnan Mo, Rirong Yang

**Affiliations:** 1 Institute of Urology and Nephrology, the First Affiliated Hospital of Guangxi Medical University, Guangxi Medical University, Nanning, Guangxi China; 2 Center for Genomic and Personalized Medicine, Guangxi Key Laboratory for Genomic and Personalized Medicine, Guangxi Collaborative Innovation Center for Genomic and Personalized Medicine, University Engineering Research Center of Digital Medicine and Healthcare, Guangxi Medical University, Nanning, Guangxi China; 3 Collaborative Innovation Centre of Regenerative Medicine and Medical BioResource Development and Application Coconstructed by the Province and Ministry, Guangxi Medical University, Nanning, Guangxi China; 4 Department of Immunology, School of Basic Medical Sciences, Guangxi Medical University, Nanning, Guangxi China; 5 Department of Urology, The Second Affiliated Hospital of Guangxi Medical University, Nanning, Guangxi China; 6 Department of Urology, The Nanning Second People’s Hospital, The Third Affiliated Hospital of Guangxi Medical University, Nanning, Guangxi China; 7 Department of Urology, The Affiliated Tumor Hospital of Guangxi Medical University, Nanning, Guangxi China

**Keywords:** Bladder, Cancer microenvironment

## Abstract

Muscle-invasive bladder cancer (MIBC) is characterized by a complex tumor microenvironment (TME) that drives aggressive progression and treatment resistance. Previous studies have highlighted the roles of cancer-associated fibroblasts (CAFs) and exhausted T (Tex) cells in MIBC, but their interactive mechanisms remain poorly understood. Here, single-cell RNA sequencing of 19 tissue samples from 12 patients—7 MIBC, 3 non-muscle-invasive bladder cancer (NMIBC), and 9 normal tissue samples—identified 13 transcriptionally distinct fibroblast clusters and 10 functionally heterogeneous T-cell subsets. Two interferon (IFN)-responsive fibroblast populations, F-ISG15 (inflammatory CAFs) and F-POSTN (myofibroblastic CAFs), were shown to predominate in the MIBC TME. In vivo experiments demonstrated that IFN-γ secreted by Tex cells polarizes CAFs to secrete CXCL12, which recruits CXCR4-expressing T cells via the CXCL12-CXCR4 chemotactic axis. Spatial analysis revealed a bidirectional loop: Tex-derived IFN-γ sustains CAF activation, whereas CAF-secreted CXCL12 amplifies Tex infiltration. Clinically, activated CAF signatures correlate with advanced disease stages and reduced patient survival in MIBC. These findings establish CXCL12 and IFN signaling as critical therapeutic targets, offering new strategies to disrupt immunosuppressive TME crosstalk and improve outcomes for MIBC patients.

## Abstract (web summary)

Single-cell RNA sequencing identifies a fibroblast gene signature promoting T-cell infiltration in muscle-invasive bladder cancer.

## Introduction

Bladder cancer (BCa) ranks tenth in incidence among cancers worldwide, with an estimated 549,000 new cases and 200,000 deaths reported in 2018^1^. Muscle-invasive bladder cancer (MIBC) accounts for 25% of newly diagnosed BCa cases and requires intensified therapeutic interventions^2^. Treatment decisions are made on the basis of clinicopathological features; however, existing staging systems exhibit limited accuracy, leading to suboptimal treatment outcomes. In recent years, various molecular subtyping systems for MIBC have been proposed but have not yet been widely incorporated into clinical practice. These classifications focus mainly on gene expression levels and may not consider the relative composition of different cell types within the tumor.

The tumor microenvironment (TME) primarily consists of tumor cells, the extracellular matrix (ECM), and numerous nontumor cells, including fibroblasts, endothelial cells, and immune cells^3^. The TME plays a crucial role in tumor maintenance and progression. With the advent of single-cell RNA sequencing (scRNA-seq) technology, our understanding of the role of fibroblasts has increased. In our previous research, three cancer-associated fibroblast (CAF) subtypes and one myogenic CAF subtype were identified^4^. Recently, exhausted T (Tex) cells have attracted increasing attention. Tex cells are a group of functionally impaired T cells commonly found in patients with chronic infections or tumors; these cells exhibit reduced effector function (indicated by features such as decreased expression of the cytotoxic molecules GZMB and GZMK) and sustained expression of inhibitory receptors (such as PD-1 and CTLA-4)^5^. These cells show changes in T-cell metabolism, impaired immune memory, and alterations in the expression of genes related to transcription and epigenetics. Despite the substantial enrichment of the CXCL12-CXCR4 signaling pathway in these CAFs, whether CAFs induce CXCL12 production in response to interferon signaling and how this affects the biological functions of Tex cells have not been determined^6^.

In this study, we collected clinical specimens to preliminarily map the single-cell landscape of BCa and analyzed the subtypes of BCa cells, fibroblasts, and T cells. We validated the subtypes of CAFs and Tex cells, with a specific focus on investigating the immune landscape of fibroblasts in MIBC and their biological functions.

## Results

### Single-cell sequencing and cell type identification

**Fig. 1:**
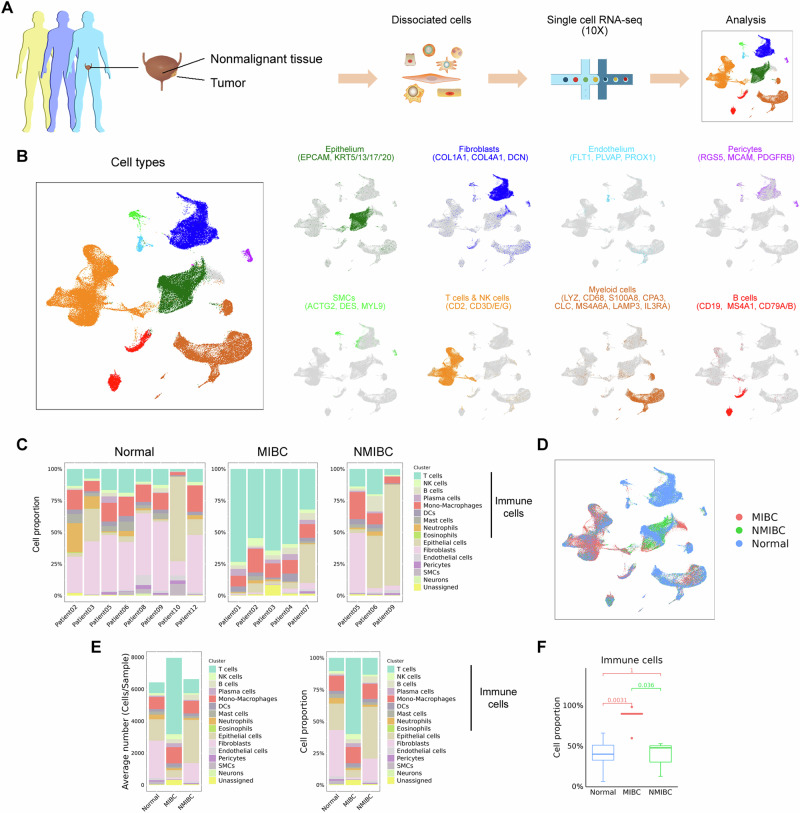
Diverse cell types in BCa delineated by scRNA-seq. **A** Schematic of the workflow. **B** UMAP plot of single cells profiled by dominant cell types (left) with defined markers (right). **C** Proportion of major cell types per patient with sample origin. **D** UMAP plot of all cells colored by sample origin. **E** Average cell numbers and cell proportions of major cell types originating from MIBC, NMIBC, and normal samples. **F** Boxplot showing the proportions of immune cells among the normal, MIBC, and NMIBC tissues.

As illustrated in the flowchart (Fig. 1A), in the first part of this study, a total of 19 tissue samples were collected from 12 patients. These samples were initially reported in our two prior studies^7,8^. The sample set comprised ten tumor samples (seven MIBC and three NMIBC samples) and nine adjacent normal tissue samples. A total of 127,391 cells, 55,721 single cells from MIBC samples, 19,887 single cells from NMIBC samples, and 51,783 single cells from adjacent nonmalignant tissue samples, were subjected to scRNA-seq.

We integrated the scRNA-seq data into a single dataset. On the basis of the expression patterns of canonical cell markers, we identified epithelial cells (normal and malignant epithelial cells), stromal cells (fibroblasts, endothelial cells, pericytes and smooth muscle cells), and immune cells [T, natural killer (NK), B and myeloid cells] as the dominant cell types (Fig. 1B). We observed that the dominant cell types were consistent across patient samples (Fig. 1C) and sample origins (Fig. 1D, Supplementary Fig. 2A). In the nonmalignant tissue samples, fibroblasts, urothelial cells, monocyte-macrophages, and T and NK cells accounted for 79.80% of the total cell population (Fig. 1E), and increased infiltration of these cell types was observed in MIBC. The proportions of immune cells in the MIBC samples were greater than those in the nonmalignant tissue samples or in the NMIBC samples (Fig. 1F), indicating different immune infiltration patterns between the MIBC and NMIBC samples. H&E and IHC staining further confirmed the differences in immune infiltration (Supplementary Fig. 2B). Taken together, our integrated data reveal the general landscape of the cells present in the BCa microenvironment and reveal differences in cell composition between tumor and nonmalignant tissue samples.

### Fibroblast subgroups in the stroma and their diverse functions in the TME

**Fig. 2:**
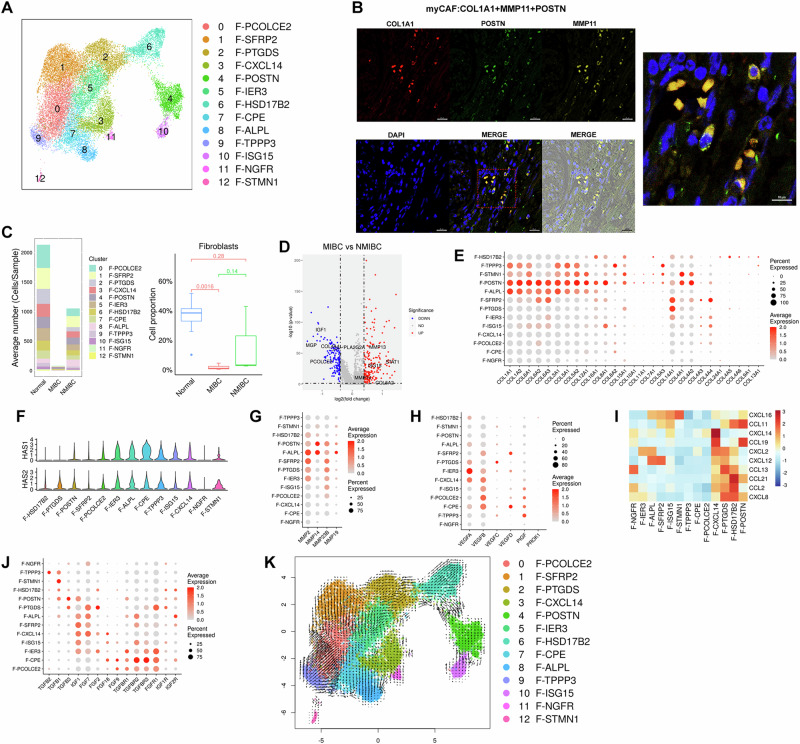
Functional specialization of the fibroblast subtypes. **A** UMAP plot showing fibroblast subtypes. **B** Representative micrograph of myCAFs in the stroma of MIBC samples immunostained for COL1A1 (red), POSTN (green) and MMP11 (yellow). Nuclei were stained with DAPI (blue). Scale bar = 20 μm, merged scale bar = 10 μm. **C** Average cell numbers and proportions of fibroblast subtypes in tissues. **D** Volcano plot showing the upregulated and downregulated genes of fibroblasts in MIBC compared with NMIBC. Red: upregulated genes; blue: downregulated genes; gray: genes whose expression did not significantly change. **E** Dot plot showing the expression levels of collagen genes. **F** Violin plot showing the expression levels of hyaluronic acid synthase. Dot plot showing the expression levels of MMP (**G**)- and angiogenesis-associated (**H**)-related genes in fibroblast subtypes. **I** Heatmap indicating the scaled expression levels of chemokine genes in fibroblast subtypes. **J** Dot plot indicating the expression levels of growth factor-related genes in fibroblast subtypes. **K** RNA velocity analysis showing the transitions of fibroblast subtypes.

We next investigated the potential function of fibroblasts in stromal microenvironment remodeling. On the basis of marker genes (Supplementary Fig. 3A), we identified 13 fibroblast subtypes among a total of 25,003 fibroblasts, which constituted 18.64% of the tissue; 4406 differentially expressed genes were used for downstream analysis (Fig. 2A). Importantly, we identified a novel fibroblast subtype called myofibroblastic cancer-associated fibroblasts (myCAFs) (Fig. 2B). The number of total fibroblasts was lower in both the MIBC and NMIBC tissues than in the normal control tissues (Fig. 2C). Compared with those in the normal and NMIBC tissues, the proportions of cluster 4 (F-POSTN) and cluster 10 (F-ISG15) cells were greater in the MIBC tissues (Fig. 2C, D), possibly due to the distinct distribution patterns of fibroblast subtypes between MIBC and NMIBC (Supplementary Fig. 3B). Among these, cluster 4 (F-POSTN) represents a fibroblast subtype characterized by high expression of the POSTN gene, which encodes a cell matrix protein. This protein is highly expressed in various tissues and organs and plays crucial roles in collagen fiber formation, muscle development, bone generation, and immune regulation^9^. Cluster 10 (F-ISG15) represents a fibroblast subtype characterized by high expression of the ISG15 gene, an interferon-induced gene that can increase the antitumor effects of the immune system, interfere with virus replication, and regulate immune responses, suggesting its potential application value in cancer immunotherapy^10^.

Recent studies have shown that transcriptionally distinct CAF isoforms exist in the TME and mainly originate from resident fibroblasts^11^. Widely reported subtypes include ECM-rich myCAFs and inflammatory CAFs (iCAFs), which are rich in transcripts associated with the inflammatory response and growth factors^12^. Collagens and hyaluronic acid are important ECM molecules in the stroma. Notably, the cells in the myCAF-(F-POSTN) and iCAF-(F-ALPL) groups expressed high levels of collagen genes, such as COL1A1/2, COL3A1, COL5A1/2, and COL6A1/2/3 (Fig. 2E). In addition, cells in the iCAF-(F-ALPL) group expressed high levels of hyaluronic acid synthases (HAS1 and HAS2) (Fig. 2F). These data suggest that fibroblasts exhibit highly context-dependent phenotypes. Furthermore, cells in the myCAF-(F-POSTN) and iCAF-(F-ALPL) groups expressed high levels of matrix metalloproteinases (MMPs; e.g., MMP2, MMP14, MMP23B, and MMP19) (Fig. 2G), indicating that these cells are involved in degradation of the basement membrane and ECM to facilitate tumor metastasis^13,14^. Moreover, cells in the iCAF-(F-CXCL14) group presented increased VEGFA and VEGFB gene expression (Fig. 2H), and the chemokine gene CXCL12 was highly expressed in cells in the iCAF-(F-ALPL) group (Fig. 2I, Supplementary Fig. 4C), indicating a proinflammatory role in recruiting immune cells into the TME. Additionally, CAFs presented increased expression of the TGFB1, IGF1, and FGF7 genes and the corresponding receptors TGFBR1/2/3, IGF1R, and FGFR1 (Fig. 2J). The RNA velocity analysis indicated that within fibroblasts, the majority of cells in cluster 10 (F-ISG15) were located at the initiation end of the transformation chain, whereas cluster 4 (F-POSTN) cells were located in the middle of the transformation chain. Moreover, most cells in cluster 10 (F-ISG15) transformed into cluster 4 (F-POSTN) cells (Fig. 2K). These data suggest that therapeutic strategies targeting neovascular endothelial cells and proangiogenic pathways could be suitable options for treating BCa.

### Enrichment of interferon-related transcription factors in fibroblast subgroups

**Fig. 3:**
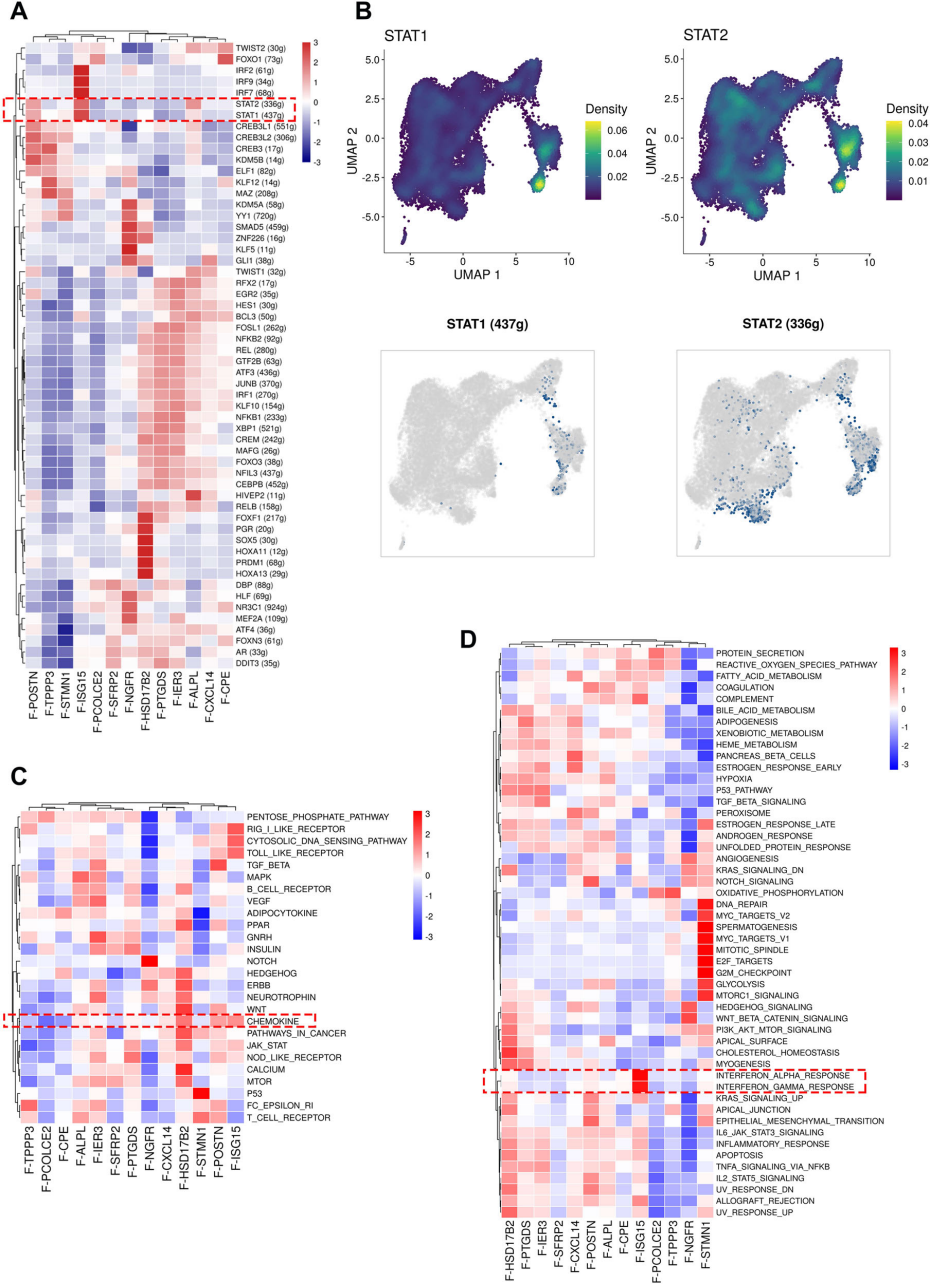
Enrichment of interferon-related transcription factors in fibroblast subgroups. **A** SCENIC gene coexpression network map. Red indicates relatively high expression, and blue indicates relatively low expression. **B** UMAP plot showing the distribution of interferon-related transcription factors in fibroblasts from different sources. The colors represent the expression levels and quantity. **C** KEGG analysis of fibroblast subtypes. Pathway enrichment results are displayed, with red indicating relatively high enrichment and blue indicating relatively low enrichment. **D** Gene set variation analysis (GSVA) of fibroblast subtypes. The functional categories are displayed, with red indicating relatively high enrichment and blue indicating relatively low enrichment.

Through single-cell regulatory network inference and clustering (SCENIC) analysis, we investigated the coexpression modules of transcription factors and potential target genes under different cellular states, aiming to identify regulons in the TME and obtain regulon activity scores. SCENIC analysis of the gene coexpression networks revealed that cluster 10 (F-ISG15), cluster 4 (F-POSTN), and cluster 8 (F-ALPL) exhibited upregulated expression of STAT1 and STAT2. Additionally, cluster 10 (F-ISG15) exhibited significantly elevated expression of interferon regulatory genes, such as IRF2, IRF7, and IRF9 (Fig. 3A). Subsequently, AUCell was employed to score the activity of each regulon in each cell to determine the activation status. This analysis revealed the activity scores and density expression patterns of the transcription factors STAT1 and STAT2 (Fig. 3B). Next, with data from 50 hallmark gene sets downloaded from the MSigDB database, pathway enrichment analysis was conducted to clarify the biological differences between fibroblast subtypes in BCa. The results revealed that fibroblasts derived from BCa patients were significantly enriched in hallmark pathways, including the interferon gamma (IFN-γ) response, interferon alpha (IFN-α) response, PI3K-Akt mTOR signaling, IL6-JAK-STAT3 signaling, epithelial‒mesenchymal transition (EMT), the inflammatory response, and TNF alpha signaling via NF-κB. The analysis revealed that the activation of cluster 10 (F-ISG15) fibroblasts was significantly associated with interferon signaling pathways, including the IFN-α and IFN-γ signaling pathways (Fig. 3C, D). These findings highlight the potential relevance of interferon signaling pathways in fibroblast subtypes. These results also provide a foundation for future studies investigating the therapeutic potential of targeting these pathways.

### T-cell subgroup dynamics and TME characteristics of MIBC

**Fig. 4:**
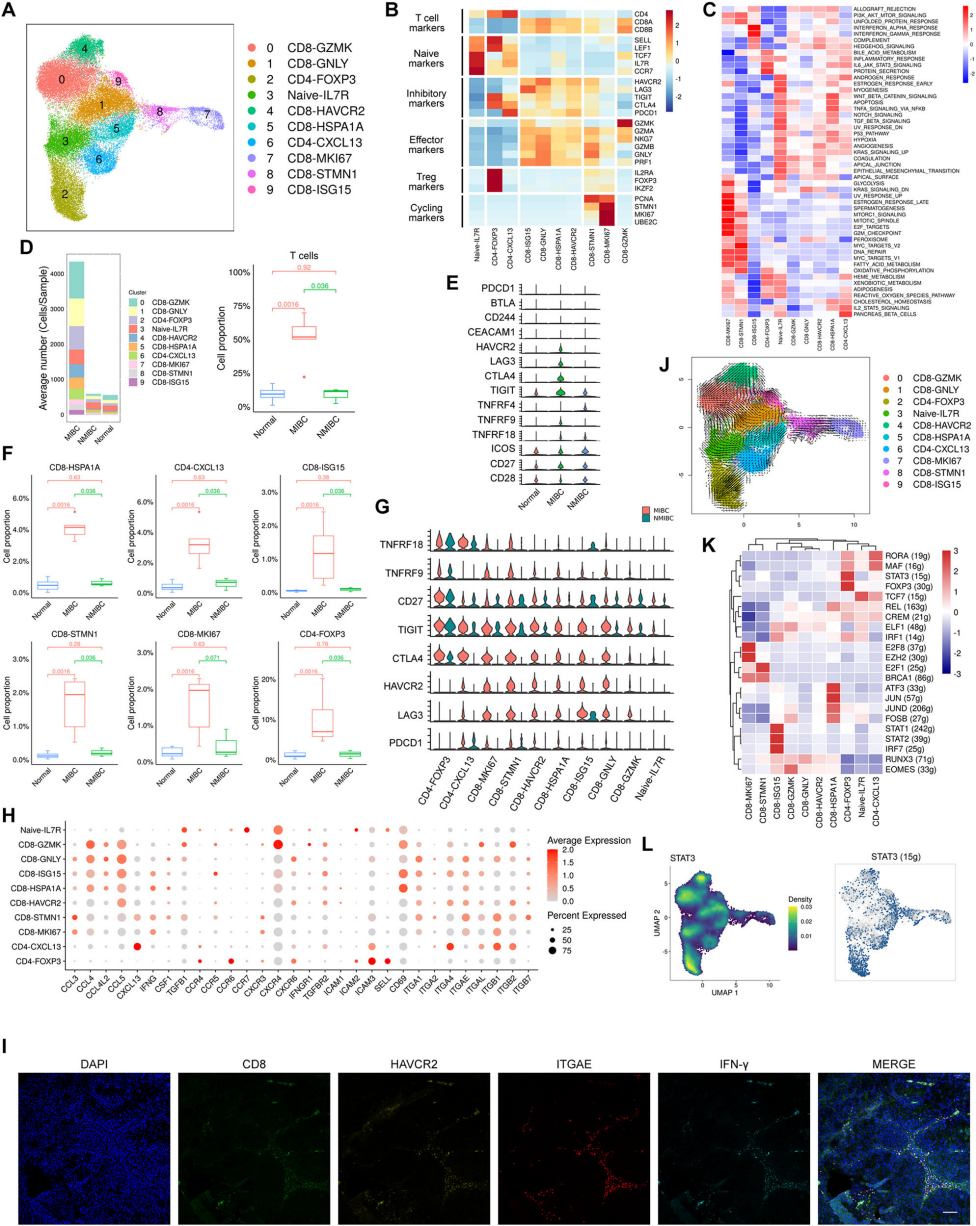
Analysis of T-cell subtypes reveals differences in the immunosuppressive microenvironment between MIBC and NMIBC. **A** UMAP plot showing T-cell subtypes. **B** Heatmap indicating the scaled expression of T-cell marker genes. **C** Heatmap showing the scaled GSVA scores of hallmark gene sets enriched in T-cell subtypes. **D** Average cell numbers and proportions of T-cell subtypes in the original samples. **E** Violin plot showing the expression levels of immune checkpoint genes in T cells from tissue samples. **F** Boxplot indicating the proportions of Tex subtypes and Tregs in MIBC. **G** Violin plot presenting the expression levels of immune checkpoint genes in T-cell subtypes between the MIBC and NMIBC samples. **H** Dot plot showing the expression levels of chemokine and chemokine receptor, cytokine and cytokine receptor, and adhesion molecule genes in T-cell subtypes. **I** Immunofluorescence staining showing that CD8 + Tex cells expressed HAVCR2, ITGAE, and IFN-γ in MIBC. Blue: DAPI; green: CD8; yellow: HAVCR2; red: ITGAE; cyan: IFN-γ. Scale bar, 100 μm. **J** Transition of T-cell subtypes on the basis of RNA velocity analysis. **K** Regulon analysis of T-cell subtypes. The rows indicate the transcription factors. The columns indicate the subtypes. Red, upregulated; blue, downregulated. **L** UMAP plot showing the expression pattern of the transcription factor STAT3 (left) and the binarized regulon activity of STAT3 (right) in T cells. The dark blue dots indicate activated regulon activity (right).

T cells are the most enriched immune cells in the TME. To elucidate the complexity of T cells in BCa, we analyzed 55,392 T cells. 10 T-cell subtypes were identified (Fig. 4A), and each subtype was annotated on the basis of marker gene expression (Fig. 4B). According to the gene enrichment analysis, an IFN-activated subtype (CD8_T-ISG15) was identified (Fig. 4A; C). The Tex group, which included CD8 T-ISG15 cells, 2 cycling subtypes (CD8-MKI67 and CD8-STMN1), CD4 Tex cells (CD4-CXCL13) and CD8 Tex cells (CD8-GNLY), was characterized by high expression of the exhaustion markers HAVCR2, LAG3, TIGIT, and CTLA4 (Fig. 4B; Supplementary Fig. 4A). Importantly, we observed high infiltration of T cells in MIBC (Fig. 4D), and T cells in MIBC presented increased expression of CTLA4, HAVCR2, LAG3, and IFN signaling-related genes compared with those in NMIBC (Fig. 4E; Supplementary Fig. 4B), suggesting differences in T-cell subtype distribution between MIBC and NMIBC. Indeed, Tex cells, as well as Tregs, were predominantly observed in MIBC (Fig. 4F; Supplementary Fig. 4C). Further analysis of immune checkpoint gene expression revealed increased expression of the inhibitory markers HAVCR2, LAG3, and CTLA4 in the Tex group of MIBC patients, whereas NMIBC patients presented elevated expression of the costimulatory molecule CD27 (Fig. 4G), indicating differences in the TME between MIBC patients and NMIBC patients. Interestingly, low expression of the programmed cell death 1 (PDCD1/PD-1) gene was observed in Tex cells (Fig. 4G), suggesting that PD-1 signaling may not be the primary pathway leading to T-cell exhaustion. We further explored the potential mechanisms by which these T-cell subgroups are recruited to the TME. The CD8_T-GZMK subgroup and pre-T subgroup expressed the CXCR4 gene, indicating their recruitment by the chemokine CXCL12 (Fig. 4H). The CD8 Tex subtype presented high levels of adhesion molecule genes, such as CD69 and ITGAE (CD103) (Fig. 4H; I; Supplementary Fig. 4D), suggesting that these cells can persist in the TME. The CD8 Tex subtype exhibited relatively high expression of chemokine genes, including CCL3/4/5, suggesting their ability to recruit other immune cells to increase immune infiltration (Fig. 4H). Moreover, both CD4 Tex and CD8 Tex cells presented elevated expression of CXCL13 (Fig. 4H; Supplementary Fig. 4D). Notably, the CD8 Tex subtype displayed increased expression of IFN-γ genes (Fig. 4H; I; Supplementary Fig. 4D), indicating that the CD8 Tex subtype primarily induces strong IFN-γ signaling in the MIBC TME. Cell trajectory analysis demonstrated that CD8 Tex cells may originate from CTLs, CD8 T-GZMK cells, or CD8 T-ISG15 cells (Fig. 4J). SCENIC analysis revealed that CD4 Tex cells, CD8 Tex cells, and Tregs presented upregulation of transcription factors, including STAT3 (Fig. 4K, L). In particular, CD8 T-ISG15 cells were enriched in MIBC (Fig. 4F), further confirming that IFN-γ signaling induces T-cell exhaustion in MIBC^15^. Collectively, our results suggest that the immunosuppressive microenvironment in MIBC is different from that in NMIBC. The proportion of Tregs increased in MIBC, and the accumulation of Tex cells with robust IFN-γ signaling dominated the MIBC immune microenvironment.

### Impact of CXCL12-CXCR4 secreted by fibroblasts on the migration, apoptosis, and cell cycle of Tex BCa cells

**Fig. 5:**
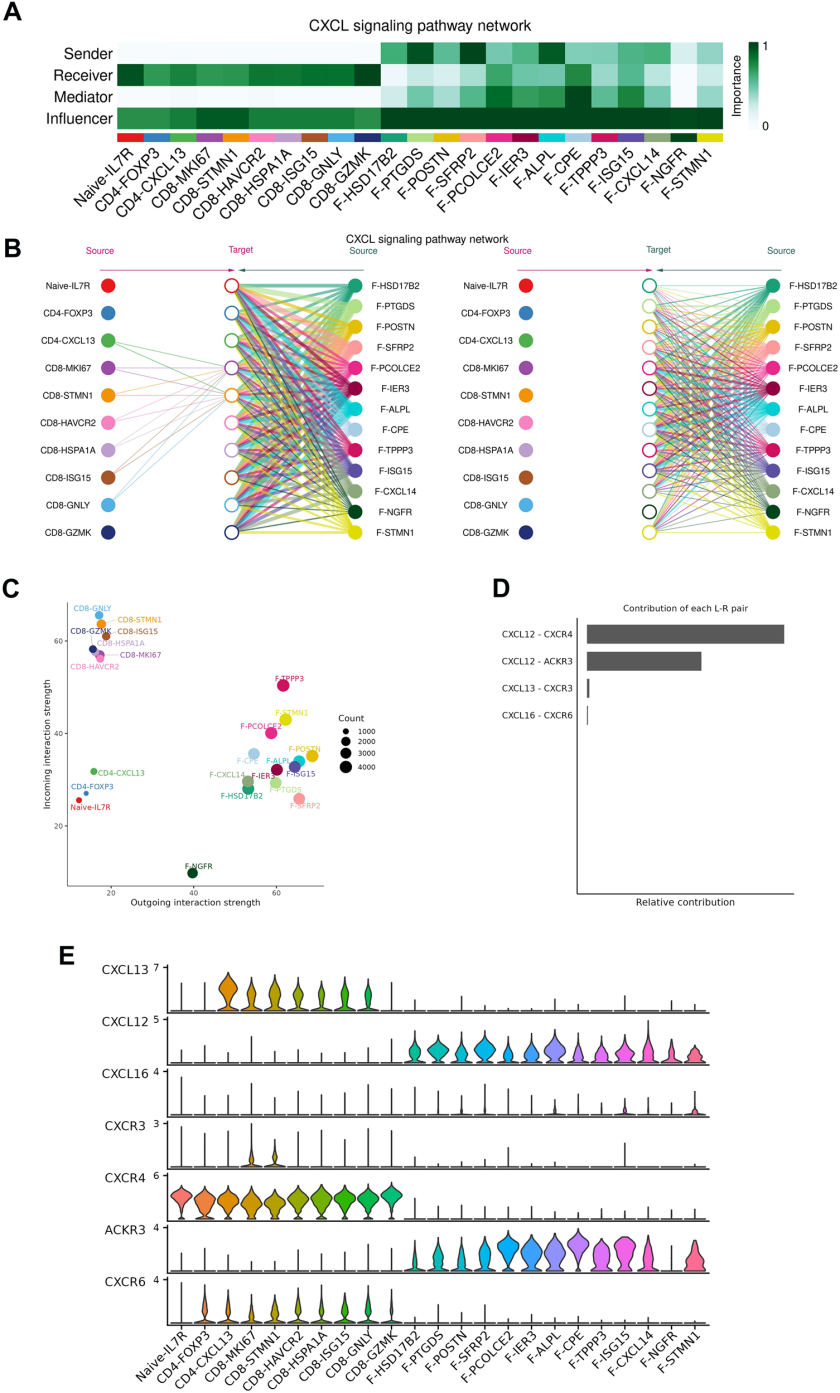
Fibroblast–T-cell interactions. **A** Role recognition diagram in the cellular network. **B** Hierarchical diagram: ‘Source’ indicates a cell group emitting signals, ‘‘Target’’ indicates a cell group receiving signals, and the circle color indicates the cell group. **C** Role analysis of two cell groups: the circle size represents the number of cells. **D** Ligand‒receptor pathway contribution diagram. **E** Distribution of genes related to chemotactic factors.

We next analyzed the interactions between fibroblasts and T cells. Through CellChat analysis of network centrality weights, we identified the roles of each cell type in recognizing two groups of cells in the CXCL signaling pathway. We found that fibroblasts act as senders, whereas T cells act as receivers (Fig. 5A). A hierarchical diagram was constructed and revealed a strong signaling role for all 13 fibroblast subgroups in the overall interaction network of the CXCL pathway.

Additionally, the seven subgroups of CD4 + and CD8 + T cells are capable of sending signals, whereas the ten T-cell subgroups are capable of receiving signals. Moreover, the 13 fibroblast subgroups not only send signals to T cells but also have autocrine signaling capabilities, indicating that fibroblasts can send signals to themselves. These findings suggest complex bidirectional communication and regulatory mechanisms between T cells and fibroblasts (Fig. 5B). The CellChat results also indicated that CAFs may act on the surface of T cells via the chemokine factor CXCL12, leading to substantial infiltration of T cells into the TME of MIBC tumors (Fig. 5C). The ligand‒receptor signaling contribution map further confirmed this conclusion (Fig. 5D). Finally, the violin plot shows high levels of CXCL12 and CXCR4 in each cell subtype of the two groups (Fig. 5E). These results suggest that in MIBC patients, fibroblasts may attract CXCR4 + T cells to the immune microenvironment through CXCL12 chemotaxis.

### Fibroblast–T-cell interactions are mediated by IFN-γ and CXCL12 in MIBC

**Fig. 6:**
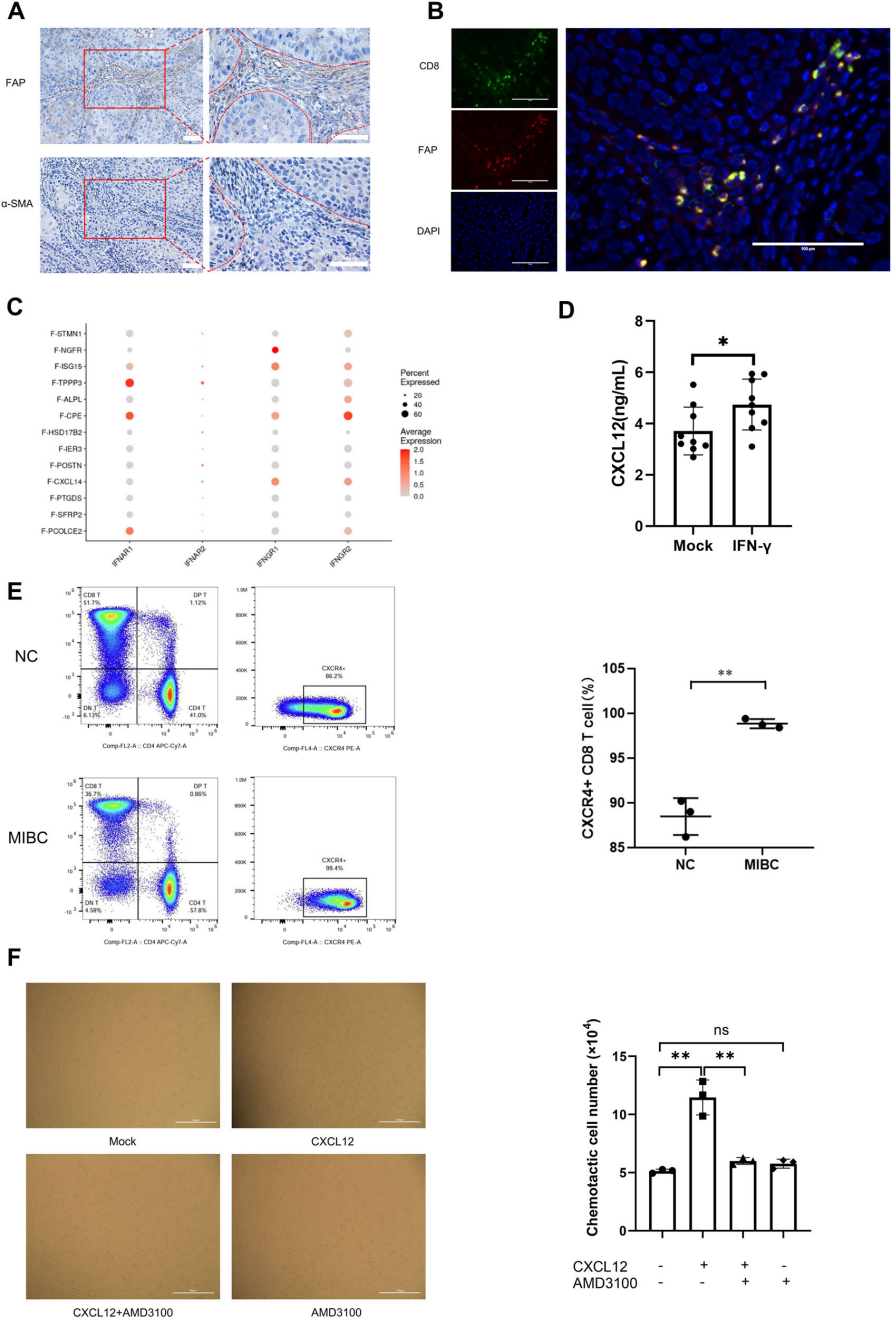
Molecular mechanisms of fibroblast-mediated regulation of T cells by IFN-γ. **A** Immunohistochemical staining showing that fibroblasts in cancer tissue samples are predominantly located in the stromal region, with a distinct boundary formed with cancer nests. The red box indicates the magnified area. Scale bar = 50 μm. **B** Immunofluorescence staining of T cells and fibroblasts from BCa samples indicating their coexistence in the stromal region. Scale bar = 100 μm. **C** Dot plot showing the expression of interferon receptors in fibroblast subtypes. The color intensity indicates the expression level of functional markers in each cell subtype, with darker red indicating higher expression. The circle size indicates the number of cells. **D** Expression of CXCL12 in the supernatant of fibroblasts stimulated with IFN-γ. *P < 0.05, **P < 0.01, ***P < 0.001, *n* = 9. **E** CXCR4 expression in peripheral blood CD8 + T cells from healthy donors (*n* = 3) and MIBC patients (*n* = 3). Chi-square test. *P < 0.05, **P < 0.01, ***P < 0.001. **F** CXCL12 chemotaxis in Jurkat T cells. ns = not significant, *P < 0.05, **P < 0.01, ***P < 0.001, *n* = 3. Scale bar = 200 μm.

FAP and α-SMA are common markers of CAFs^16^. We conducted immunohistochemical staining and immunofluorescence staining on MIBC tissues. The histological results suggest that in tissues from patients with MIBC, the number of FAP + fibroblasts is significantly greater in the stroma than in the tumor region, whereas the number of α-SMA + fibroblasts is not significantly different (Fig. 6A). Our immunofluorescence labeling results revealed close proximity between T cells and fibroblasts, indicating the interaction between T cells and fibroblasts (Fig. 6B). In MIBC tissue, IFN-γ was expressed across all fibroblast types in the stromal region but at different levels, and IFNGR1 and IFNGR2 were the main interferon receptor expressed in these fibroblast subpopulations (Fig. 6C). Further enzyme-linked immunosorbent assays (ELISAs) revealed a significant increase in CXCL12 secretion by fibroblasts under IFN-γ stimulation (Fig. 6D), indicating that IFN-γ promotes the release of the chemotactic factor CXCL12 by fibroblasts. We subsequently assessed the expression levels of CXCR4 in peripheral blood CD8 + T cells from MIBC patients. The findings revealed that, compared with that in healthy donors, CXCR4 expression in peripheral blood CD8 + T cells from MIBC patients was significantly elevated (Fig. 6E, *P* < 0.05). Finally, we assessed the phenotypes of the Jurkat T cells and found that 69.7% of these cells were CXCR4 + (Supplementary Fig. 5A). Functional experiments revealed that CXCL12 induces the migration of CXCR4 + Jurkat T cells, suggesting that fibroblasts promote the migration of CXCR4 + T cells to the stroma by secreting abundant CXCL12, thus contributing to a more complex immune microenvironment in BCa (Fig. 6F, *P* < 0.05). In addition, flow cytometry revealed that CXCL12 did not significantly affect the apoptosis or the cell cycle of Jurkat T cells after 12, 24, or 48 h of stimulation (Supplementary Fig. 5B; Supplementary Fig. 5C, *P* > 0.05).

### Fibroblast-associated gene signatures as prognostic markers in MIBC

**Fig. 7:**
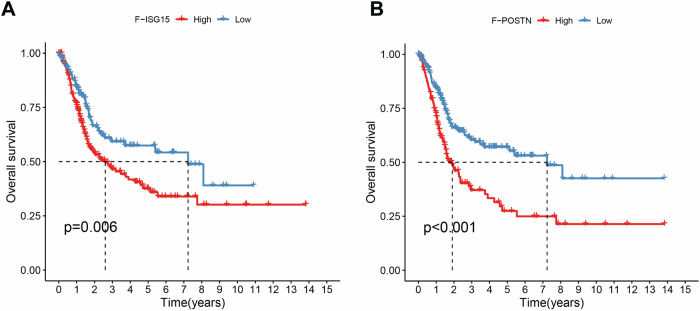
Prognostic value of fibroblast subtypes in MIBC. **A** The Kaplan‒Meier method was used to analyze the effect of the ISG15 expression level on the survival rate of patients with MIBC (*P* < 0.05). **B** The Kaplan‒Meier method was used to analyze the effect of the POSTN expression level on the survival rate of patients with MIBC (*P* < 0.001).

To determine the impact of various fibroblast subtypes on the survival prognosis of patients with MIBC, we utilized the TCGA database and conducted LASSO regression analysis to select characteristic genes associated with fibroblasts for Kaplan‒Meier analysis. We also employed LASSO regression analysis to screen fibroblast-related genes that can predict the survival outcomes of patients with MIBC infiltration (Fig. 7A, B). The LASSO model included 13 fibroblast-related genes, and univariate Cox regression analysis revealed that 11 genes, including ten groups (F-ISG15, F-POSTN), KCNN3, CXCL12, CCDC80, IGF1, SERPINF1, LAMA2, SVEP1, LHFPL6, PTGER3, COL18A1, and ITGBL1, were risk genes (hazard ratio > 1). These findings suggest that fibroblasts have an adverse effect on the survival of patients with MIBC (Supplementary Fig. 6).

## Discussion

BCa is the most common tumor of the male urinary system and ranks among the top ten most prevalent malignant tumors globally. Despite major progress in the surgical and chemotherapeutic treatment of BCa, effective treatments, especially for MIBC, are urgently needed^17^. Therefore, in-depth research on the TME of MIBC is urgently needed to identify new therapeutic targets. The TME consists of tumor cells, structural cells, immune cells, and the ECM^18^. In this study, we collected tissue samples from 12 BCa patients, seven MIBC tissues, three NMIBC tissues, and nine normal tissues, and generated a single-cell atlas of BCa. Notably, bladder tissue samples were obtained from full-thickness longitudinal sections from the inner wall to the outer wall, providing a comprehensive depiction of the TME. Integration of the data revealed the major cell types present in the BCa TME. We focused particularly on the subtyping of CAFs and T cells, validating the subgroups of CAFs and Tex cells.

The TME is a multicellular system characterized by complex interactions between tumor and stromal components^13,19^. As one of the most abundant stromal components in the TME, CAFs play crucial roles in mediating tumor initiation, progression, and immune suppression^14^. Building upon these concepts, we focused on exploring the diversity of CAFs and understanding their various roles in regulating the biological aspects of the TME. Chen et al.^20^ performed scRNA-seq on 8 BCa patients and identified fibroblast subgroups, stratified as iCAFs or myCAFs on the basis of the expression of PDGFRα and RGS5. In this study, a comparison of the differentially expressed genes revealed distinct functions of the two CAF subgroups, suggesting that iCAFs may promote tumorigenesis through functions related to migration, proliferation, and angiogenesis. By analyzing the 50 genes most significantly differentially expressed among the 13 fibroblast subgroups, we identified the cluster 4 (F-POSTN) and cluster 10 (F-ISG15) genes as significantly expressed genes. UMAP analysis and RNA velocity analysis indicated that there may be a transformation relationship between cluster 10 (F-ISG15) and cluster 4 (F-POSTN) cells. Our results also indicated that iCAFs highly expressed the chemokine CXCL12, suggesting its role in recruiting immune cells expressing CXCR4 to the TME. Both iCAFs and myCAFs expressed IFN receptors, and the high expression of ISG15 in specific subgroups suggested the strong involvement of IFN in regulating fibroblasts. Strong IFN signaling is a prominent feature of the TME of MIBC, and high concentrations of IFN-γ promote the formation of an immunosuppressive TME^21^. High levels of IFN-γ may induce Tex cells, which are characterized by reduced sensitivity to antigens and inactivated immune cell functions, potentially weakening the immune system’s effective response against tumors^22^. A study by Ma et al.^23^ revealed a new subgroup of BCa fibroblasts whose formation was induced by IFN signals; these cells also expressed the urea transporter SLC14A1. While activation of the WNT5A pathway in tumor cells can promote tumor stemness and mediate chemotherapy resistance, our research focused mainly on the CXCL signaling pathway activated by IFN signaling in fibroblasts. We performed sc-RNA-seq and experimental verification and assessed crosstalk with T cells to investigate the interaction between T cells and CAFs in the TME of MIBC. One of the key functions of CAFs is to deposit and modify the fibrous ECM, promoting tumor cell invasion^24^. This process results in various biomechanical consequences, including increased contractile strength, tissue tension, and increased tumor hardness. This increased stiffness triggers further activation of CAFs, forming a self-sustaining positive feedback loop. Our results revealed that the myCAF (F-POSTN) cluster significantly expressed MMP-14, indicating that myCAFs disrupt the ECM and bladder basement membrane, supporting this viewpoint. Using multi-immunofluorescence technology, we verified the expression of MMP-11 and POSTN by myCAFs, as well as the expression of CXCL14 by iCAFs, in MIBC specimens. These findings provide additional support for the roles of these CAF subtypes in the TME.

We also performed subtyping of T cells and identified two CD4 + T-cell subgroups, seven CD8 + T-cell subgroups, and one naive cell subgroup. The dynamic interplay between antitumor immunity and immune evasion is a constant feature in the process of tumor formation and development. CD8 + T cells typically differentiate into a functionally exhausted phenotype after prolonged exposure to tumor antigens, but this is a dynamic and partially reversible process^25^. This process involves extensive phenotypic switching and continuous intermediate states, making it challenging to define the exhaustion level of the functional state of T cells fully and accurately. Tex cells exhibit high expression of multiple inhibitory receptors, such as PD1, PD-L1, TIM-3, TIGIT, and CTLA-4, and alterations in transcription factors, including T-cell factor (TCF1) and thymocyte selection-associated HMG BOX (TOX)^26^. Additionally, Tex cells exhibit hierarchical dysfunction of processes related to cellular effectors, namely, the production of IFN-γ, TNF, and IL-2. Zhang et al.^27^ established a comprehensive outline of Tex-specific pathways, including the TNF, IL-2, IFN-γ, and T-cell cytotoxic pathways. These researchers proposed an immunotyping scheme based on Tex through pan-cancer analysis. In our analysis of MIBC, we observed significant infiltration of T cells; compared with those in NMIBC, T cells in MIBC presented increased expression of genes associated with immune checkpoints [such as CTLA4, HAVCR2 (TIM-3), LAG3] and IFN signaling pathways. This finding suggests a shift in the immune landscape between MIBC and NMIBC that may support more aggressive immune evasion mechanisms and increased infiltration of exhausted T-cell subsets in MIBC^28^. Furthermore, in the context of BCa, T-cell infiltration appears to be quite prominent. The high proportion of T cells in BCa tissues may indicate an ongoing attempt by the immune system to control tumor progression. However, the upregulation of exhaustion markers such as PD-1, CTLA-4, and TIM-3 in MIBC further suggests that T cells may undergo functional impairment despite their high abundance. This result indicates that complex interplay between immune activation and immune suppression occurs within the TME.

Tex cells express CXCR4 and IFN-γ, indicating that Tex cells can be recruited to the TME by CXCL12, which is secreted by iCAFs, and may regulate the transformation of fibroblast subtypes^29^. In this study, SCENIC analysis revealed that Tex cells are regulated mainly by the inhibitory transcription factor STAT3. STAT3 is expressed mainly in Tregs and Tex cells, and its target genes are also highly expressed in Tregs and Tex cells, suggesting that STAT3 is activated and functional in Tex cells. Immunohistochemistry and immunofluorescence validation experiments revealed high concentrations of Tex cell-secreted IFN-γ in the MIBC microenvironment. Bioinformatics and sc-RNA-seq analyses revealed that iCAFs act on the CXCR4 receptor on the surface of T cells through the chemokine factor CXCL12, promoting the infiltration of many T cells into the TME of MIBC. Moreover, Tex cells can regulate the function of fibroblast subgroups through IFN-γ signaling. The bubble chart illustrates the strength of the interactions between Tex cell subgroups and CAF subgroups, suggesting that T cells are attracted primarily by iCAFs, whereas CAF subgroups are regulated mainly by the IFN-γ signal from Tex cells.

In previous studies, the proportion of CAFs was consistently negatively correlated with prognosis. We found that a set of 11 characteristic genes of CAFs was correlated with the overall survival of patients with MIBC using TCGA transcriptomic data and clinical information; thus, this gene signature has prognostic value. We believe that CAFs are promising therapeutic targets for the treatment of MIBC. Although our research strongly contributes to the clarification of the heterogeneity of CAFs in MIBC and their interaction with T cells, this study still has some limitations. First, our experiments were conducted primarily at the in vitro level, and we plan to further validate the chemotaxis mechanisms through in vivo studies in future research. Second, despite the emphasis on the regulatory effect of CAFs on the CXCL12–CXCR4 pathway mediated by IFN-γ activation, further research is needed to determine its impact on other relevant signaling pathways. In the future, we plan to perform integrated single-cell spatial transcriptomic and proteomic analysis for a comprehensive understanding of spatial information and functionality in MIBC, providing support for more in-depth exploration.

## Conclusion

In this study, comprehensive sc-RNA-seq and functional experiments were performed for a detailed exploration of the heterogeneity of CAFs in MIBC and their pivotal role within the TME. Our findings not only help elucidate CAF diversity but also provide a robust theoretical framework for the development of innovative therapeutic strategies targeting CAFs. In future studies, we will aim to further elucidate the intricate interaction network between CAFs and immune cells in the MIBC TME, facilitating the development of more effective and personalized treatment strategies.

## Methods

### Human tumor specimens

The specimens were obtained from the Department of Urology at the First Affiliated Hospital of Guangxi Medical University and the Affiliated Tumor Hospital of Guangxi Medical University. All patients who participated in this study provided informed consent. The study received approval from the Medical Ethics Committee of Guangxi Medical University (Ethical Approval Numbers: 2018-003 and 2022-106). All ethical regulations relevant to human research participants were followed. Tumor samples were collected from 8 patients. Among these 8 patients, 5 (P01, P02, P03, P04, and P07) presented with MIBC, and 3 (P05, P06, and P09) presented with NMIBC. Nonmalignant bladder tissue samples were collected from nine patients (P02, P03, P05, P06, P08, P09, P10, P11, and P12) and samples from three patients (P10, P11, and P12) were analyzed in our previous study (Supplementary Table 1).

### Tissue processing and preparation of single-cell suspensions

These steps were described in our previous study^4^. Full-thickness samples of the tumor and adjacent nonmalignant tissues were generated with a surface diameter of 1 cm (Supplementary Fig. 1). Briefly, fresh biopsy samples from BCa tissues were cut into small pieces and rinsed with phosphate-buffered saline (PBS; Gibco, USA). The tissues were then cut into 2–4 mm fragments via sterile scissors, washed, and resuspended twice. The tissue samples were subjected to 30 min of digestion at 37 °C in digestion solution [1 mg/mL collagenase I (Gibco, 5401020001) and 1 mg/mL DNase I (Roche, 10104159001) in HBSS]. The digestion process was terminated with DMEM (WISENT, 319-006-CL) containing 10% FBS (Gibco, 10099141). A 70 μm cell strainer (Falcon) was subsequently employed to filter out large tissue fragments. Red blood cells (RBCs) were eliminated via incubation of the samples with RBC lysis buffer (10× diluted to 1×; BioLegend, 420301) for 5 min on ice. The samples were further filtered through a 40 μm cell strainer (Falcon). Viable cells were counted via trypan blue staining (Gibco, 15250-061); the included samples had more than 80% viable cells. All living cells were directly subjected to scRNA-seq.

### Preparation and sequencing of the 10x Genomics library

The concentration of the single-cell suspensions was adjusted to 1500 cells/µL, and 22,000 cells/sample were loaded onto a Chromium Controller using Chromium Single-Cell 3′ Reagent Kits (v3 chemistry). Sequencing was performed on an Illumina Nova S6000 instrument.

### scRNA-seq data processing

scRNA-seq data were processed and quantified via the Cell Ranger (3.1.0) pipeline (https://support.10xgenomics.com/single-cell-gene-expression/software/pipelines/latest/using/count) with ‘--id --transcriptome --fastqs --localcores’ arguments. First, the GRCh38 reference used to align the reads was obtained from 10x Genomics. An overview website and a file containing a feature table, barcode table, and feature–barcode matrix were generated. The website provides a summary of sample information, including the estimated number of cells, mean reads per cell, median genes per cell, median unique molecular identifier (UMI) counts per cell, and sequencing saturation (Supplementary Table 1). The feature–barcode matrix was transformed into a Seurat object via the R package Seurat (version 3.1.1)^30^. Low-quality cells were excluded on the basis of specific criteria: mitochondrial gene percentage >15%, <300 genes/cell, and >7500 genes/cell for tissue samples or >5000 genes/cell for PBMC samples. Cells meeting the following criteria were retained: mitochondrial gene percentage <15% and 300 < genes/cell < 4000. After filtering, the feature‒barcode matrix for each sample underwent normalization via the “NormalizeData” function with default parameters (“LogNormalize” method and scale.factor = 10,000). The top 2000 highly variable genes (HVFs) were identified via the “FindVariableFeatures” function (“vst” method). The feature–barcode matrix was subsequently scaled via the “ScaleData” function, and doublets for each sample were subsequently identified via the R package DoubletFinder, which assumes a 5% doublet formation rate^31^.

### Sample aggregation, dimensionality reduction, and clustering

After the doublet cells were removed, the filtered feature‒barcode matrices of all the samples were merged. For removal of batch effects across different samples, the anchor correspondences of the merged data were identified with the “FindIntegrationAnchors” function, and the first 50 dimensions were used for calculation. These computed anchors were subsequently used for the integration of the merged data with the “IntegrateData” function. The integrated data were normalized, and the HVFs were identified via Seurat. The variables “percent.mito” and “nCount_RNA” were regressed out with the “ScaleData” function, and principal component analysis (PCA) was performed via the “RunPCA” function. The top 50 principal components were used for dimensionality reduction to visualize cells with the “RunUMAP” function, and the major clusters were identified with the “FindNeighbors” and “FindClusters” functions with a resolution of 1.4 to obtain relatively good results. Notably, cells expressing dual-lineage genes were excluded from downstream analysis.

### Analysis of functional cell subtypes within the major cell clusters

50 major clusters were identified by mapping canonical marker genes. For identification of subtypes within each major cluster, the major cell clusters were subclassified and reclustered. The procedures included normalization, HVF identification, data scaling, PCA calculations, batch effect removal with the “harmony” method, UMAP visualization, and cluster identification^32^.

### Identification of cluster marker genes

Differential gene expression analysis was performed using the Seurat “FindAllMarkers” function; marker genes for major clusters or cell subtypes detected in at least 25% of the cells were sorted according to the mean log2 (fold change) value and filtered according to a minimum log2 (fold change) value of 0.25. A gene was considered significantly differentially expressed when the adjusted *p* value was <0.05 (adj.*p*.). Biological process enrichment analysis was performed for the top 50 differentially expressed genes in each major cluster or subtype via Metascape^33^.

### GuangxiSCENIC analysis

For analysis of the regulatory characteristics of cell clusters, SCENIC was employed to analyze gene regulatory networks as previously described^34^. Briefly, coexpression modules were inferred via the “runGENIE3” function, and potential direct binding targets (regulons) of transcription factors were identified via the human motif database of 10 kb around the transcription start site (TSS) for RcisTarget. The regulon activity in the cell clusters was subsequently evaluated via AUCell and averaged. A heatmap for each regulon cluster was generated with the R package heatmap.

### RNA velocity analysis

The velocyto.R program was used for RNA velocity analysis^35^. First, spliced/unspliced reads for each sample were annotated with velocyto.py via the possorted_genome_bam.bam file, which was generated via Cell Ranger and then saved in a .loom file. Second, the .loom files for each sample were loaded into R and combined to generate count tables that contained spliced and unspliced reads. Next, the cells in the bottom 0.5% of the total unspliced transcript count were filtered out. Third, genes whose average spliced variant expression was lower than 0.2 or whose average unspliced variant expression was lower than 0.05 in at least one cluster were removed. Finally, arrows indicating RNA velocity information were added to the UMAP plot obtained from Seurat.

### Cell‒cell interaction analysis

Ligand‒receptor (L–R) interaction scores were calculated according to the R package CellChat^36^. L‒R pairs representing interactions between B cells and T-cell subtypes are presented according to interaction scores.

### Immunofluorescence, immunohistochemistry, and hematoxylin and eosin (H&E) staining

In brief, the sections were deparaffinized, rehydrated, and washed with PBS (P1010-2, Solarbio), and antigen retrieval was then performed via a high-pressure heat repair process with sodium citrate buffer (catalog number C1032; Solarbio). Endogenous peroxidase activity was blocked with 3% H_2_O_2_ for 30 min. After incubation with blocking buffer (PBS with 10% goat serum) at room temperature for 15 min, the sections were incubated with primary antibodies [COL1A1 (NBP1-77457AF647, Novus, USA), POSTN (1:200, LS-C442376, LifeSpan, USA), MMP11 (1:700, Ab119284, Abcam, USA), CD8 (1:100, Ab237709, Abcam, USA), HAVCR2 (CST, 45208, USA), ITGAE (Ab24202, Abcam, USA), IFN-γ (Ab231036, Abcam, USA), FAP (Ab207178, Abcam, USA), α-SMA (Ab8211, Abcam, USA), CD45 (1:500, Ab8216, Abcam, USA), CXCL14 (1:200, LS-C481321, LifeSpan, USA), and PLA2G2A (1:200, Ab23705, Abcam)] in a humidified chamber at 4 °C in the dark overnight, washed three times, and stained with a secondary antibody in a humidified chamber at 4 °C in the dark. For immunohistochemistry staining, an HRP-conjugated secondary antibody was used, and the sections were reacted with diaminobenzidine (DAB) prior to counterstaining with hematoxylin. For immunofluorescence, nuclear staining was performed with DAPI, and the sections were mounted with antifade mounting medium. For H&E staining, the sections were dewaxed and hydrated, and the nuclei and cytoplasm were directly stained with H&E. The sections were subsequently sealed with neutral gum. Images were acquired using a Leica Microsystems microscope (DMI8), a Leica SP8 microscope for fully integrated Stimulated emission depletion (STED) microscopy, or a NanoZoomer S60 digital slide scanner (C13210, Hamamatsu, Japan).

### ELISA

IMR90 and MRC-5 human fibroblasts and Jurkat T cells were obtained from the Type Culture Collection of the Chinese Academy of Sciences (Shanghai, China). The cells were divided into two groups for experiments: the mock group and the 20 ng/mL IFN-γ stimulation group. Logarithmic growth phase cells, at a density of 5 × 10^4^ cells per well, were seeded in a 48-well cell culture plate, and after overnight adhesion, they were treated as indicated above for 6‒8 h.

During the ELISA experiment, the reagent kit was equilibrated at room temperature for 30 min before the test. The supernatant of the stimulated cells was centrifuged and stored. The experiments included blank, standard, and test sample wells. Different concentrations of standard were added to the standard wells, and diluted samples were added to the test sample wells. The enzyme mixture was added to each well, and the plate was shaken and cultured at 37 °C for 60 min. After washing, color reagents A and B were added to each well, and color development was carried out in the dark at 37 °C for 15 min. The absorbance (OD value) was measured via an enzyme marker within 15 min after the reaction was terminated.

### Transwell migration assay

For the cell migration experiments, Jurkat T cells at a density of 7.5 × 10^5^ cells/mL were seeded in a culture plate and cultured in medium containing 100 ng/mL CXCL12 (HY-P7287, PeproTech, USA). The cell suspensions were placed in the upper and lower chambers of a Transwell plate, and after 15 h of culture, the migrated cells were collected and counted. ImageJ counting was used to analyze the numbers of nonmigrated and migrated cells, providing a deeper understanding of the effect of cell chemotaxis.

### Flow cytometry

For flow cytometric staining of cell markers, isolated PBMCs were first resuspended in blocking buffer and incubated with a CXCR4 antibody (306,505, BioLegend, USA). Next, the cells were washed and incubated with an Alexa Fluor® 488-conjugated secondary antibody (Abcam, UK). The Annexin V-FITC/PI Apoptosis Kit and Cell Cycle Staining Kit (Beyotime, China) were used to determine the proportions of apoptotic cells (including cells in early and late stages of apoptosis) and the cell cycle distribution of Jurkat T cells. Finally, the stained cells were analyzed using an Accuri™ C6 Plus flow cytometer (BD, USA), and the data were processed with FlowJo software (version 10.8.1).

### Survival analysis

The “survival analysis” module of Gene Expression Profile Interaction Analysis version 2 (GEPIA2) (http://gepia2.cancer-pku.cn/) was used to construct Kaplan‒Meier curves for The Cancer Genome Atlas (TCGA) Bladder Urothelial Carcinoma (BLCA) cohort.

### Statistics and reproducibility

R (version 4.1.0) was used for scRNA-seq data analysis, and GraphPad Prism (version 8.0) was used for graphical processing. The in vivo experiments were independently repeated at least three times. Statistical analyses between two groups were performed using an unpaired two-tailed Student’s *t* test, and comparisons among multiple groups were conducted using one-way ANOVA in GraphPad Prism 8.0. The following criteria were used to determine statistical significance: *P < 0.05, **P < 0.01, ***P < 0.001, ****P < 0.0001, and ns indicates no significant difference.

### Reporting summary

Further information on research design is available in the Nature Portfolio Reporting Summary linked to this article.

## Supplementary information

Supplementary information

Description of additional supplementary materials

Supplementary data 1

Reporting summary

## Data Availability

The transcriptomes reported in this paper are publicly available through Dryad 10.5061/dryad.nzs7h450j and Supplementary Data 1. All the relevant data are available from the corresponding author upon reasonable request.

## Code availability

All the code used to perform the analysis is available at github https://github.com/Zachary-zigeliu/Mo-s-Lab.git.
