# Supplementary material for: Single-cell RNA sequencing reveals a fibroblast gene signature that promotes T-cell infiltration in muscle-invasive bladder cancer
Source: Commun Biol. 2025 May 3;8:696. doi: 10.1038/s42003-025-08094-9 (PMC12049545; doi:10.1038/s42003-025-08094-9)
Supplement: Supplementary file 1 — Supplementary information [file 42003_2025_8094_MOESM1_ESM.pdf]

Supplementary Information

Supplementary Table 1: Patient information

Patient	Gender	Age year	Sample	Tumor Stage	Cell Number	Mean Reads per Cell	Medium Gene per Cell	Medium UMI per Cell	Sequncing Saturation	After Filtering	Features	History of treatment
1	Male	66	Tumor	pT2aN0M0	9956	104123	1820	5588	73	7749	22388	Treatment naïve
2	Male	63	Normal		5601	121416	2177	6549	71	4463	21391	Treatment naïve
			Tumor Site1	pT2bN0M0	10198	104706	2310	6262	59.7	7334	22960	
			Tumor Site2	pT2bN0M0	10209	97438	2217	5758	62	7883	22701	
3	Male	64	Normal		11236	100272	3359	13240	57.6	7784	23087	Treatment naïve
			Tumor Site1	pT2aN0M0	11692	109441	2634	7458	69	10971	22812	
			Tumor Site2	pT2aN0M0	13455	94411	2521	6971	63.6	12654	22841	
4	Male	54	Tumor	pT2aN0M0	9543	107841	1853	5362	76.9	7625	22182	Treatment naïve
5	Male	74	Normal		9815	96989	2667	12018	64.1	8255	22578	Treatment naïve
			Tumor	pT1N0M0	8378	96873	2864	11974	64.2	6860	22277	
6	Male	57	Normal		6861	69248	2642	9705	57.3	5400	21462	Treatment naïve
			Tumor	pT1N0M0	11890	70598	2591	10362	41.4	7558	22935	
7	Male	51	Tumor	pT3aN1M0	8301	98631	1552	5929	57.6	4438	22197	Treatment naïve
8	Male	63	Normal		5946	99613	2492	9512	73.4	4266	20842	Treatment naïve
9	Female	70	Normal		6845	88566	3246	12675	67.3	5719	21745	Treatment naïve
			Tumor	pT1N0M0	11545	66810	3293	15139	48.1	6516	21934	
10	Female	35	Normal		7856	77459	1094	3045	88.6	6748	19549	Treatment naïve
11	Male	48	Normal		487	173733	1666	4963	91	409	15380	Treatment naïve
12	Male	37	Normal		12662	26189	1208	3578	71.3	11463	18976	Treatment naïve

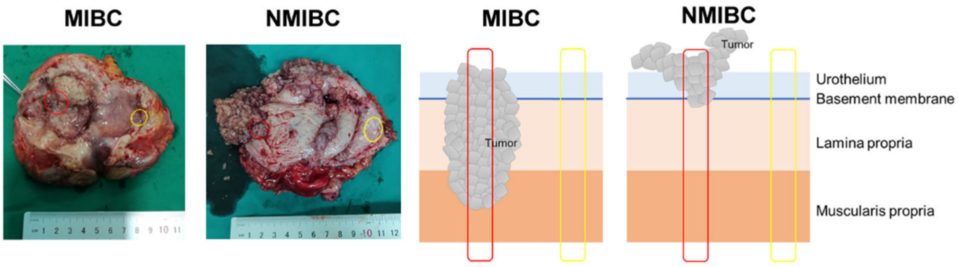

Supplementary Figure 1. Schematic diagram of sample collection

The schematic diagram shows how full-thickness samples of tumors and adjacent nonmalignant tissues were generated. The harvested tumor sample is indicated by the red circle and box, and the harvested adjacent nonmalignant tissue is indicated by the yellow cycle and box.

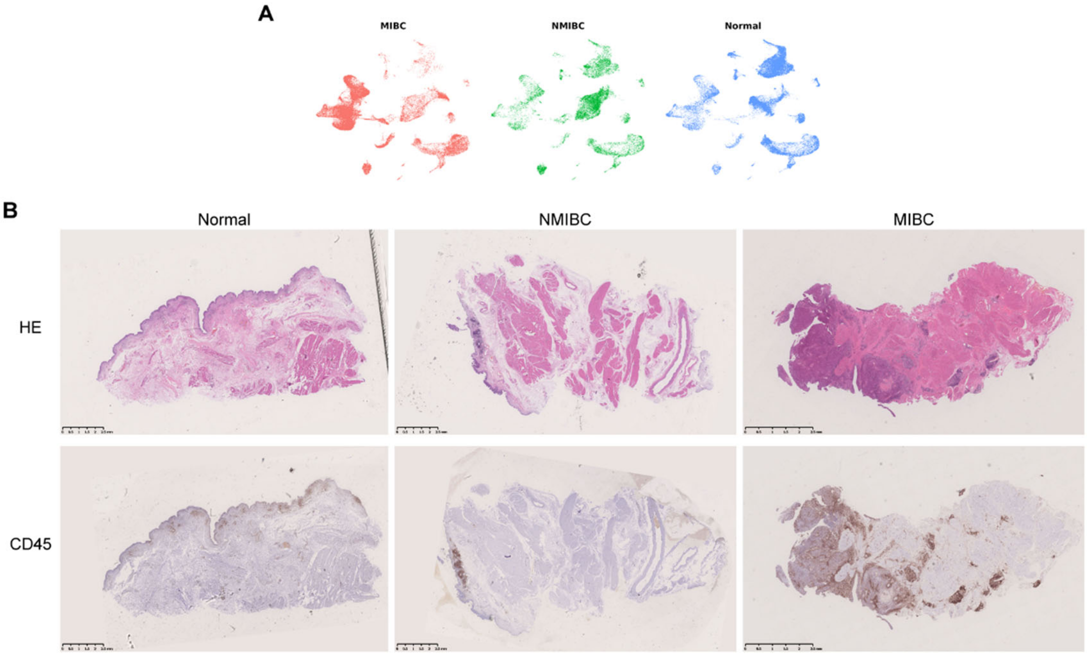

Supplementary Figure 2. Sample origin of all cells and immune infiltration of bladder tissues

(A) UMAP plot showing cells originating from MIBC, NMIBC, and normal samples. Each dot indicates an individual cell.

(B) H&E and IHC staining showing immune infiltration in normal, NMIBC, and MIBC tissues.

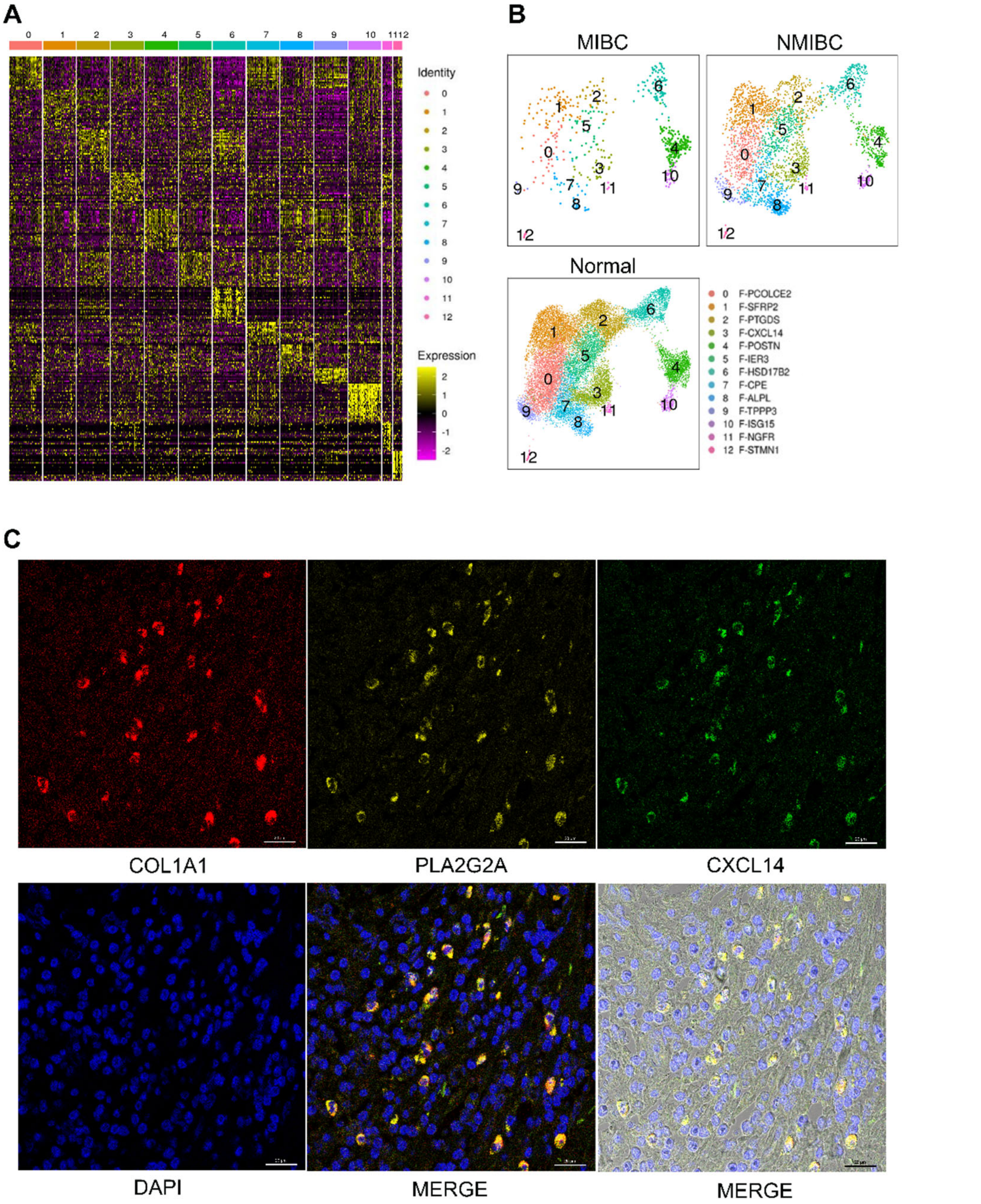

Supplementary Figure 3. Characterization of fibroblast subtypes and identification of iCAF-CXCL14 cells

(A) Heatmap presenting the marker genes for fibroblast subtypes.

(B) UMAP plots showing the sample origins of fibroblasts.

(C) Immunofluorescence staining of novel markers of fibroblast subtypes. Blue: DAPI; green: CXCL14; yellow: PLA2G2A; red: COL1A1. Scale bar, 20 μm.

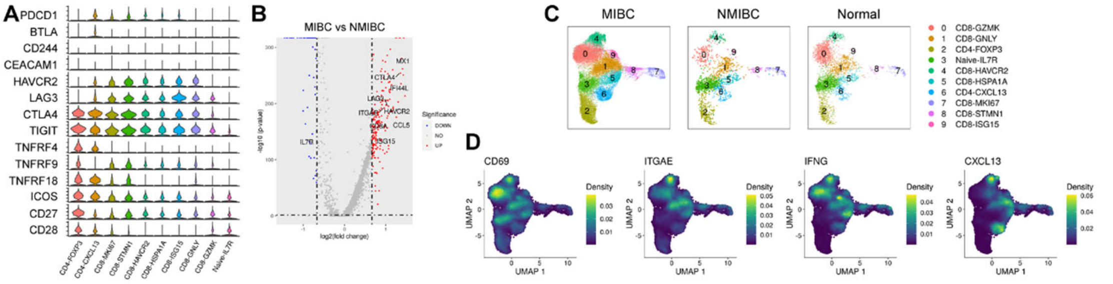

Supplementary Figure 4. Analysis of T cells

(A) Violin plot showing the expression levels of immune checkpoint genes in T-cell subtypes.

(B) Volcano plot showing the genes whose expression was upregulated or downregulated in T cells from MIBC patients compared with those from NMIBC patients. Red: upregulated genes; blue: downregulated genes; gray: genes whose expression did not significantly change.

(C) UMAP plots showing the sample origin of T cells.

(D) UMAP plot showing the expression densities of the CD69, ITGAE, IFNG, and CXCL13 genes in T-cell subtypes.

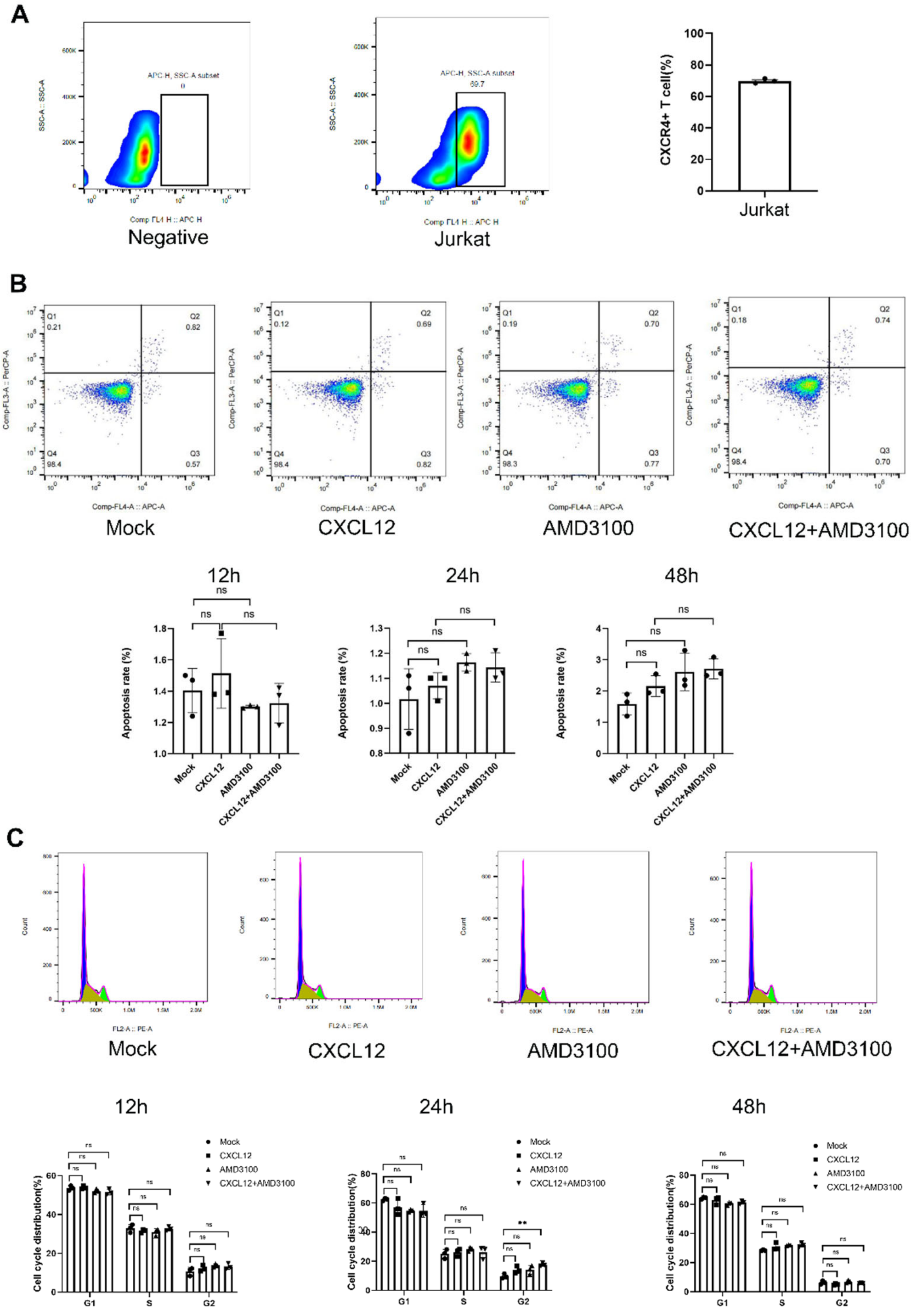

Supplementary Figure 5. Analysis of CXCR4 expression and the impact of CXCL12 treatment on Jurkat T cells

(A) Flow cytometry analysis showing the expression of CXCR4 on Jurkat T cells. n = 3.

(B) Effects of CXCL12 and AMD3100 on the distribution of apoptotic Jurkat T cells after 12, 24, and 48 hours of stimulation. **P* < 0.05, ***P* < 0.01, ****P* < 0.001, n = 3.

(C) Effects of CXCL12 and AMD3100 on the cell cycle distribution of Jurkat T cells after 12, 24, and 48 hours of stimulation. **P* < 0.05, ***P* < 0.01, ****P* < 0.001, n = 3.

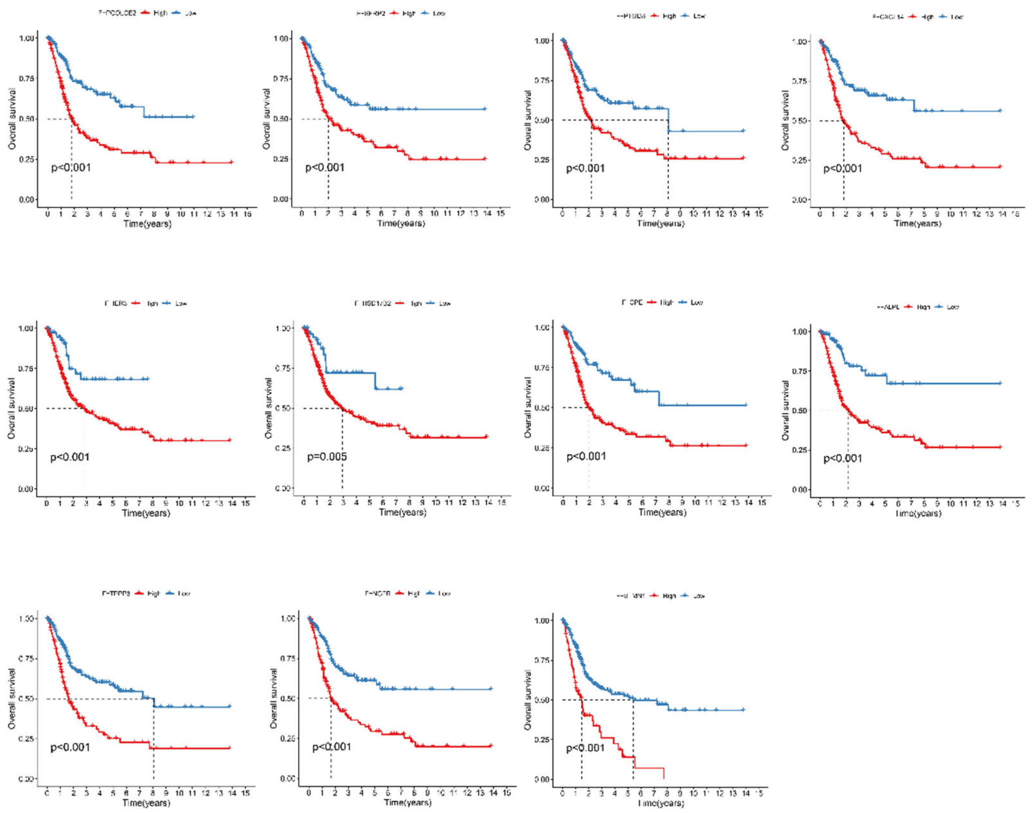

Supplementary Figure 6. Prognostic value of fibroblast subtypes in MIBC

The Kaplan–Meier method was used to analyze the effects of the infiltration levels of different fibroblast subtypes on the survival rate of patients with MIBC (*P*<0.05).

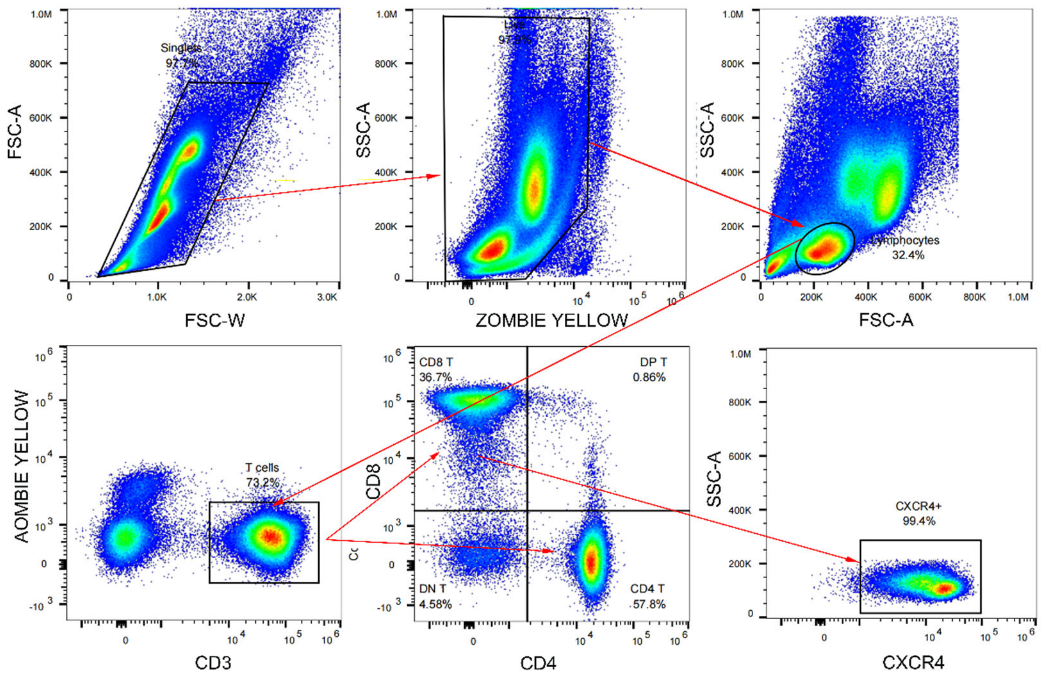

The gating strategy used for CXCR4+CD8+T cells from human MIBC tumors.
