# Supplementary material for: Single-cell RNA sequencing reveals a fibroblast gene signature that promotes T-cell infiltration in muscle-invasive bladder cancer
Source: Commun Biol. 2025 May 3;8:696. doi: 10.1038/s42003-025-08094-9 (PMC12049545; doi:10.1038/s42003-025-08094-9)
Supplement: Supplementary file 2 — Description of additional supplementary materials [file 42003_2025_8094_MOESM2_ESM.docx]

**Description of Additional Supplementary Files**

**File name:** Supplementary Data 1

**Description:** Data for Figure S5: Details underlying Figure S5 are provided. These data are likely to facilitate further exploration and understanding of the phenomena depicted in this supplementary figure.

Data for Figure 6D (ELISA): ELISA data presented in Figure 6D are included. This dataset can support in - depth analysis of the biochemical parameters and relationships investigated using the ELISA technique.

Data for Figure 6F (Transwell Migration Assay): Data obtained from the Transwell migration assay, as shown in Figure 6F, are available. These data are crucial for evaluating cell migration capabilities, a key aspect of the study.

Data for Figure 6E: The dataset relevant to Figure 6E is incorporated, enabling comprehensive examination of the data - driven insights presented in this figure.
