# Supplementary material for: Single-cell RNA sequencing reveals a fibroblast gene signature that promotes T-cell infiltration in muscle-invasive bladder cancer
Source: Commun Biol. 2025 May 3;8:696. doi: 10.1038/s42003-025-08094-9 (PMC12049545; doi:10.1038/s42003-025-08094-9)
Supplement: Supplementary file 4 — Reporting summary [file 42003_2025_8094_MOESM4_ESM.pdf]

nature portfolio

Corresponding author(s): Rirong Yang

Last updated by author(s): 2025/3/13

Reporting Summary

Nature Portfolio wishes to improve the reproducibility of the work that we publish. This form provides structure for consistency and transparency in reporting. For further information on Nature Portfolio policies, see our Editorial Policies and the Editorial Policy Checklist.

Statistics

For all statistical analyses, confirm that the following items are present in the figure legend, table legend, main text, or Methods section.

n/a	Confirmed	
☐	☒	The exact sample size (*n*) for each experimental group/condition, given as a discrete number and unit of measurement
☐	☒	A statement on whether measurements were taken from distinct samples or whether the same sample was measured repeatedly
☐	☒	The statistical test(s) used AND whether they are one- or two-sided<br>*Only common tests should be described solely by name; describe more complex techniques in the Methods section.*
☐	☒	A description of all covariates tested
☐	☒	A description of any assumptions or corrections, such as tests of normality and adjustment for multiple comparisons
☐	☒	A full description of the statistical parameters including central tendency (e.g. means) or other basic estimates (e.g. regression coefficient) AND variation (e.g. standard deviation) or associated estimates of uncertainty (e.g. confidence intervals)
☐	☒	For null hypothesis testing, the test statistic (e.g. F, t, r) with confidence intervals, effect sizes, degrees of freedom and P value noted<br>*Give P values as exact values whenever suitable.*
☒	☐	For Bayesian analysis, information on the choice of priors and Markov chain Monte Carlo settings
☒	☐	For hierarchical and complex designs, identification of the appropriate level for tests and full reporting of outcomes
☒	☐	Estimates of effect sizes (e.g. Cohen's *d*, Pearson's *r*), indicating how they were calculated

Our web collection on statistics for biologists contains articles on many of the points above.

Software and code

Policy information about availability of computer code

Data collection	We used R (version 4.1.0) to collect the data. The custom code will be made available to the editors and reviewers upon request.
Data analysis	All the statistical analyses and graph generation were performed in R (version 4.1.0) and GraphPad Prism (version 8.0).

For manuscripts utilizing custom algorithms or software that are central to the research but not yet described in published literature, software must be made available to editors and reviewers. We strongly encourage code deposition in a community repository (e.g. GitHub). See the Nature Portfolio guidelines for submitting code & software for further information.

Data

Policy information about availability of data

All manuscripts must include a data availability statement. This statement should provide the following information, where applicable:

- Accession codes, unique identifiers, or web links for publicly available datasets
- A description of any restrictions on data availability
- For clinical datasets or third party data, please ensure that the statement adheres to our policy

The datasets generated and/or analyzed during the current study are not publicly available owing to limitations of ethical approval involving patient data and anonymity but are available from the corresponding author upon reasonable request.

Research involving human participants, their data, or biological material

Policy information about studies with human participants or human data. See also policy information about sex, gender (identity/presentation), and sexual orientation and race, ethnicity and racism.

Reporting on sex and gender	For analysis, participants were categorized into male and female.
Reporting on race, ethnicity, or other socially relevant groupings	This study does not involve the reporting or analysis of race, ethnicity, or other socially relevant groupings.
Population characteristics	Participants were diagnosed with either muscle-invasive bladder cancer (MIBC) or non-muscle-invasive bladder cancer (NMIBC). The study included 12 patients, with 7 patients having MIBC and 5 patients having NMIBC.
Recruitment	Participants were recruited from the Department of Urology at the First Affiliated Hospital of Guangxi Medical University and the Affiliated Tumor Hospital of Guangxi Medical University. All participants were provided with informed consent forms. No significant self-selection bias was identified in the recruitment process.
Ethics oversight	This study was approved by the Medical Ethics Committee of Guangxi Medical University (Ethical Approval Numbers: 2018-003 and 2022-0106).

Note that full information on the approval of the study protocol must also be provided in the manuscript.

Field-specific reporting

Please select the one below that is the best fit for your research. If you are not sure, read the appropriate sections before making your selection.

☒ Life sciences ☐ Behavioural & social sciences ☐ Ecological, evolutionary & environmental sciences

For a reference copy of the document with all sections, see nature.com/documents/nr-reporting-summary-flat.pdf

Life sciences study design

All studies must disclose on these points even when the disclosure is negative.

Sample size	Sample size was determined based on previous studies in the field. A total of 12 patients were included in the study, which was deemed sufficient to observe the effects of the treatment based on historical data.
Data exclusions	No data were excluded from the analyses. All participants' data were included in the final analysis.
Replication	The study was conducted with sufficient replicates to verify reproducibility, and all attempts at replication were successful.
Randomization	Participants were assigned to treatment groups based on pre-established criteria. Randomization was not performed due to the nature of the study, as participants were selected based on specific inclusion criteria.
Blinding	Blinding was not applicable in this study.

Reporting for specific materials, systems and methods

We require information from authors about some types of materials, experimental systems and methods used in many studies. Here, indicate whether each material, system or method listed is relevant to your study. If you are not sure if a list item applies to your research, read the appropriate section before selecting a response.

Materials & experimental systems

n/a	Involved in the study	
☐	☒	Antibodies
☒	☐	Eukaryotic cell lines
☒	☐	Palaeontology and archaeology
☒	☐	Animals and other organisms
☐	☒	Clinical data
☒	☐	Dual use research of concern
☒	☐	Plants

Methods

n/a	Involved in the study	
☒	☐	ChIP-seq
☐	☒	Flow cytometry
☒	☐	MRI-based neuroimaging

Antibodies

Antibodies used	"Anti-αSMA antibody (Abcam, catalog #Ab8211) was used in the study."<br>"Anti-IFN-γ antibody (Abcam, catalog #Ab231036) was used in the study."<br>"Anti-COL1A1 antibody (Novus, catalog #NBP1-77457AF647) was used in the study."<br>"Anti-POSTN antibody (LifeSpan, catalog #LS C442376) was used in the study."<br>"Anti-MMP11 antibody (Abcam, catalog #Ab119284) was used in the study."<br>"Anti-CD8 antibody (Abcam, catalog #Ab237709) was used in the study."<br>"Anti-FAP antibody (Abcam, catalog #Ab207178) was used in the study."<br>"Anti-CD4P antibody (Abcam, catalog #Ab8216) was used in the study."<br>"Anti-POSTN antibody (LifeSpan, catalog #LS C481321) was used in the study."<br>"Anti-ITGAE antibody (Abcam, catalog #Ab24202) was used in the study."<br>"Anti-HAVCR2 antibody (CST, catalog #45208) was used in the study."<br>"Anti-PLA2G2A antibody (Abcam, catalog #Ab23705) was used in the study."
Validation	All antibodies used in this study have been validated by the manufacturers for use in the respective applications.

Clinical data

Policy information about clinical studies

All manuscripts should comply with the ICMJE guidelines for publication of clinical research and a completed CONSORT checklist must be included with all submissions.

Clinical trial registration	This study did not involve a clinical trial and therefore does not have a clinical trial registration number.
Study protocol	This study did not follow a clinical trial protocol, as it is not a clinical trial.
Data collection	Data were collected retrospectively from patient records at the First Affiliated Hospital of Guangxi Medical University and the Affiliated Tumor Hospital of Guangxi Medical University, and no prospective clinical data were collected for this study.
Outcomes	This study did not define primary or secondary outcomes as the focus was on single-cell analysis and validation.

Plants

Seed stocks	This study did not involve the use of plant materials or seed stocks.
Novel plant genotypes	This study did not involve the generation of novel plant genotypes.
Authentication	This study did not involve the authentication of plant materials.

Flow Cytometry

Plots

Confirm that:

☒ The axis labels state the marker and fluorochrome used (e.g. CD4-FITC).

☒ The axis scales are clearly visible. Include numbers along axes only for bottom left plot of group (a 'group' is an analysis of identical markers).

☒ All plots are contour plots with outliers or pseudocolor plots.

☒ A numerical value for number of cells or percentage (with statistics) is provided.

Methodology

Sample preparation	For flow cytometry, PBMC were isolated from peripheral blood of healthy donors and resuspended in blocking buffer. The cells were stained with CXCR4 antibody, followed by an Alexa Fluor® 488-conjugated secondary antibody.
Instrument	Flow cytometry data were collected using an Accuri™ C6 Plus flow cytometer (BD Biosciences, USA).
Software	Flow cytometry data were analyzed using FlowJo software (version 10.8.1).

Cell population abundance	The abundance of CXCR4+CD8+ T cells among CD8+ T cells is 99.4%.
Gating strategy	The gating strategy for flow cytometry was based on forward scatter (FSC) and side scatter (SSC) to identify viable cells. Cells were first gated on FSC vs SSC to exclude debris and doublets. The positive and negative populations were defined based on the fluorescence intensity of the target marker, such as CD8+ T cells. The gating was further refined using appropriate isotype controls.

☒ Tick this box to confirm that a figure exemplifying the gating strategy is provided in the Supplementary Information.
